# Opposite regulation by PI3K/Akt and MAPK/ERK pathways of tissue factor expression, cell-associated procoagulant activity and invasiveness in MDA-MB-231 cells

**DOI:** 10.1186/1756-8722-5-16

**Published:** 2012-07-11

**Authors:** Chaoquan Hu, Limin Huang, Caroline Gest, Xiaodong Xi, Anne Janin, Claudine Soria, Hong Li, He Lu

**Affiliations:** 1 INSERM, U728, F-75010, Paris, France; 2 Université Paris Diderot, Sorbonne Paris Cité, Laboratoire de pathologie, UMR-S 728, F-75010, Paris, France; 3 DIFEMA, Merci (EA 3829), Faculté de Médecine et de Pharmacie, Université de Rouen, 76183, Rouen, France; 4 Shanghai Institute of Hematology, Shanghai Rui Jin Hospital, Shanghai Jiao Tong University School of Medicine, 200025, Shanghai, China; 5 AP-HP-Hôpital Saint-Louis, Laboratoire de pathologie–Paris, F-75010, Paris, France

**Keywords:** Breast cancer, Tissue factor, Gene expression regulation, MAPK/ERK pathway, PI3K/Akt pathway, Pro-coagulation activity, Tumor invasiveness

## Abstract

**Background:**

Tissue factor (TF), an initiator of blood coagulation, participates in cancer progression and metastasis. We recently found that inhibition of MAPK/ERK upregulated both full length TF (flTF) and soluble isoform TF (asTF) gene expression and cell-associated TF activity in breast cancer MDA-MB-231 cells. We explored the possible mechanisms, especially the possible interaction with EGFR and PI3K/Akt pathways.

**Methods:**

A plasmid containing TF promoter −2174 ~ +128 plus luciferase reporter gene was introduced into MDA-MB-231 cells to evaluate TF promoter activity. In order to study the interaction of these pathways, ERK inhibitor (PD98059), PI3K inhibitors (LY294002, wortmannin), Akt inhibitor (A6730), and EGFR inhibitor (erlotinib) as well as the corresponding siRNAs were used to treat MDA-MB-231 cells, and ovarian cancer OVCAR-3 and SKOV-3 cells. Quantitative PCR and western blot were used to determine TF expression. One stage clotting assays were used to measure pro-coagulation activity of the MDA-MB-231 cells.

**Results:**

We show that PI3K inhibitors LY294002, wortmannin and A6730 significantly inhibited TF promoter activity, and reduced TF mRNA and protein levels due to the inhibition of Akt phosphorylation. In contrast, ERK inhibitor PD98059 and ERK siRNA enhanced TF promoter activity by 2.5 fold and induced an increase in TF mRNA and protein levels in a dose dependent manner in these cells. The PI3K/Akt pathway was shown to be involved in PD98059-induced TF expression because the induction was inhibited by PI3K/Akt inhibitors. Most interestingly, the EGFR inhibitor erlotinib and EGFR siRNA also significantly suppressed PD98059- or ERK siRNA-induced TF promoter activity and TF protein expression. Similar results were found with ovarian cancer cells SKOV-3 and OVCAR-3. Furthermore, in MDA-MB-231, mRNA levels of asTF were regulated in a similar way to that of TF in response to the cell treatment.

**Conclusions:**

This study showed a regulatory mechanism in which MAPK/ERK signals inhibit EGFR/PI3K/Akt-mediated TF expression in breast cancer MDA-MB-231 cells. The same regulation was observed in ovarian cancer OVCAR-3 and SKOV-3 cells. Interestingly, we observed that both flTF and asTF could be regulated in a parallel manner in MDA-MB-231. As the PI3K/Akt pathway and EGFR regulate TF expression in cancer cells, targeting these signaling components is expected to potentially inhibit TF expression-associated tumor progression.

## Background

Tissue factor (TF), a key molecule required to initiate blood coagulation, is essential for embryo development, maintenance of vascular integrity and tissue repair [1-4]. It is widely expressed on cells of extravascular compartments and initiates hemostasis upon tissue injury. TF expression is a complicated and finely regulated process and studies are still ongoing because the regulatory mechanisms of TF expression are diversified in different cells such as endothelial cells and cancer cells. Recent studies have demonstrated that the binding of transcription factors to the TF promoter region and the binding of miRNAs to its 3’ UTR region both regulate TF expression [5,6].

A variety of cancer cells, such as breast cancer cells, show aberrant high levels of TF expression. In colorectal carcinoma cell lines, the activation of K-ras oncogene and inactivation of p53, result in high levels of TF expression in a manner dependent on MEK/mitogen-activated protein kinase (MAPK) and phosphatidylinositol 3-kinase (PI3K) [7]. TF overexpression was also reported to be due to amplified and active EGFR and depended mostly on activation of JunD/AP-1 complex through PI3K/Akt and JNK pathways [8]. The inhibition of TF promoter activity by the ERK inhibitors U0126 and PD98059 had been observed in breast cancer cells and LPS-stimulated human monocytic cells [9,10]. Obviously, the roles of MAPK and PI3K pathways depend on the species involved, particular stimuli and the interaction of signal pathways [11]. It is now well accepted that TF on tumor cells initiates PAR2-dependent signaling with subsequent effects on tumor growth and simultaneously induces thrombin generation that facilitates metastasis [4].

The TF gene is composed of 6 exons and mature TF is a trans-membrane protein of 263 amino acids (flTF). When alternative TF pre-mRNA splicing occurs, exon 5 is excluded during mRNA transcription and the transmembrane domain and C-terminal cytoplasmic domain of TF are replaced by a different 40 amino acid peptide, resulting in a shorter, 206 aa soluble TF (alternatively spliced, asTF) [12,13]. The synthesis of asTF is determined by the binding of specific Serine/arginine Rich (SR) Proteins to the exonic splicing enhancer (ESE) within exon 5 [14]. The concentration of asTF mRNA was reported to be about 30 fold lower than flTF in endothelial cells and the inhibition of PI3K/Akt reduced asTF mRNA in these cells [15]. Moreover, in addition to its potential role in thrombogenesis, asTF binds to β1 and β3 integrins and induces angiogenesis [16]. Recent studies also indicated that asTF can stimulate tumor angiogenesis by its binding to integrins [17,18]. Clinical data showed that asTF was an indicator of poor prognosis in lung cancer patients [18,19].

Given the importance of tissue factor on cancer cells, this study focused on the roles of PI3k/Akt and MAPK/ERK in the regulation of TF expression in MDA-MB-231 cells, especially the signaling crosstalk between the MAPK/ERK and PI3K/Akt pathways. We also studied the effects of TF expression on the activation of coagulation and cell invasiveness, one of the critical steps of tumor metastasis.

## Methods

### Cell lines and chemicals

Human breast cancer epithelial cell lines MDA-MB-231, human ovarian epithelial cell lines SKOV-3 and OVCAR-3 were obtained from the American Type Culture Collection (Rockville, MA, USA). Cells were cultured in Dulbecco's modified Eagle’s medium(DMEM) supplemented with 10% fetal bovine serum (FBS), 1% L-glutamine, and 1% sodium pyruvate (all from Invitrogen, USA) at 37°C in a humidified atmosphere containing 5% CO_2_. To determine the roles of MAPK/ERK and PI3K/Akt in TF expression, PD98059 (Calbiochem, San Diego, USA), a selective inhibitor of MAPK/ERK kinase (MEK); LY294002 (Sigma-Aldrich, USA) and wortmannin (Sigma-Aldrich, USA), PI3K inhibitors; an Akt1/2 inhibitor A6730 (Sigma-Aldrich, USA); erlotinib (TARCEVA, Genentech, USA), an inhibitor of EGFR; an anti-EGFR antibody cetuximab (Merck Serono, Switzerland), Akt siRNA(s659, s660), ERK siRNA(s11140, s11141), EGFR siRNA(s564, s565) and scrambled oligonucleotide (Applied biosystem, CA, USA) were used to treat the cells for 24 h [19]. siRNA transfection was performed with INTERFERin (Polyplus-transfection SA, France) with the mixture of 2 siRNAs (2 × 15 nM of each siRNA) to knockdown one gene.

### TF promoter activity analysis

**Figure 1:**
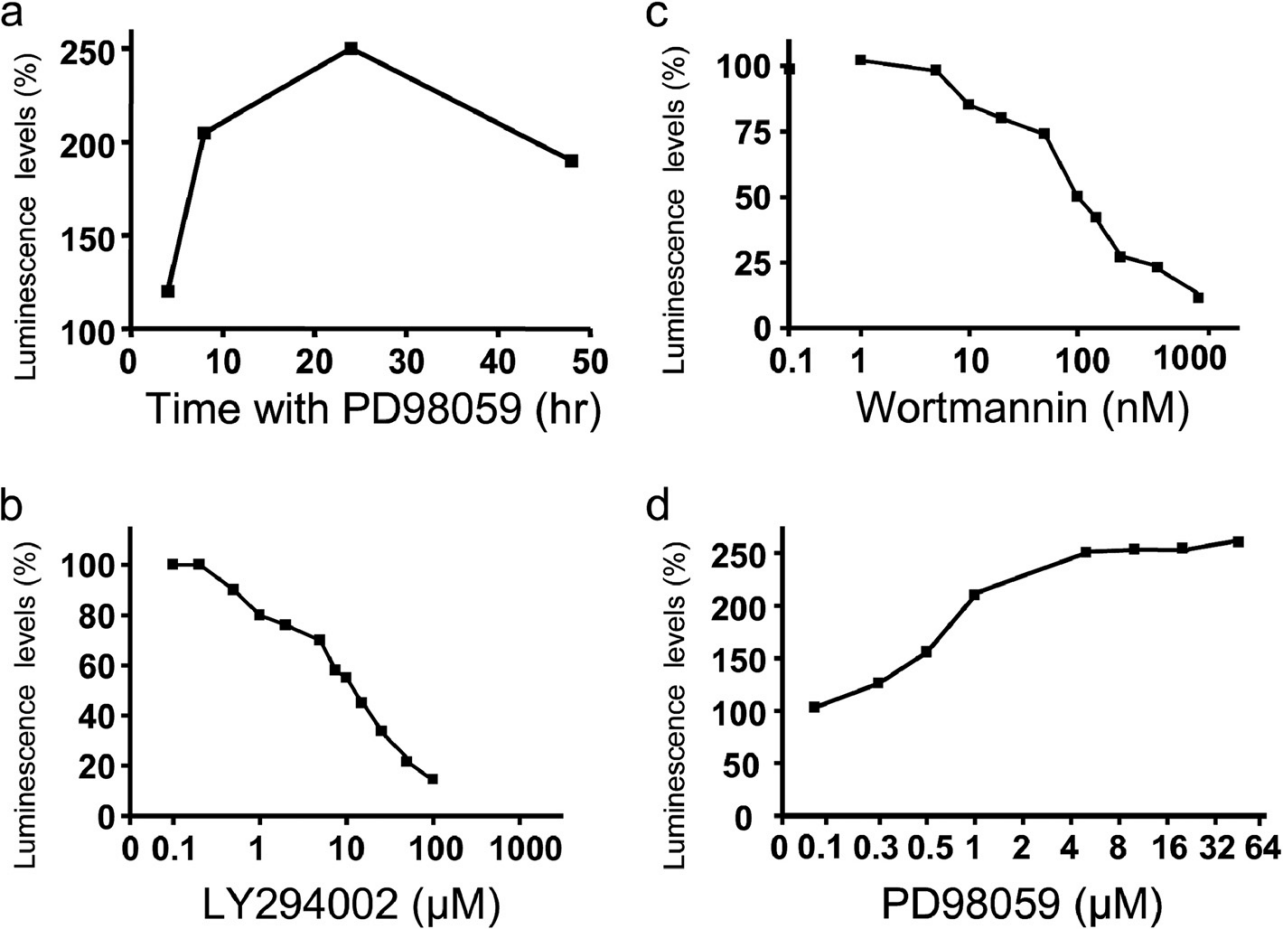
**TF promoter activity in MDA-MB-231-TFluc cells treated by LY294002, wortmannin and PD98059. Panel a:** MDA-MB-231-TFluc cells were treated by PD98059 at 10 μM for different time periods, then washed. **Panels b-c:** the cells were treated by LY294002 (b), wortmannin (c) and PD98059 (d) at indicated concentrations for 24 hrs. The activity of luciferase in the cells was measured. The data represent the means values of three independent experiments.

MDA-MB-231 cells at 30%–40% confluence were transfected with a constructed plasmid pGL4-TFluc, carrying firefly luciferase reporter gene under the control of the promoter of human TF within −2174 ~ +128[20]. Transfected cells were then selected by hygromycin (Invitrogen, USA) at the concentration of 400 μg/ml. Survived clones of the cells were screened for bioluminescence in the complete media supplemented with luciferase assay system (Promega, USA) in FLUOstar Optima Microplate Reader (Germany). The established cell line MDA-MB-231-TFluc was used for evaluating the TF gene expression. After the treatment with the agents at the indicated concentrations and time periods, the harvested cells were washed, counted with trypan blue exclusion assay to check the cell viability. The bioluminescence of the samples was then quantified for TF promoter activity. In our experiments, the cells gave maximal luminescence level at 24–48 h after the treatment (Figure 1a).

### Quantitative polymerase chain reaction (qPCR) assay

The treated or non-treated cells were harvested and total RNA was prepared by SV total RNA isolation system kit (Promega, USA). The purity of total RNA were checked by a ratio of A260/A280 (>1.9). Total RNA (50 ng) was used to synthesize cDNA in 20 μl reaction solution using a kit of GoScript Reverse Transcription System (Promega, USA). Then 2 μl of cDNA was used for qPCR assay in triplicates with taqman® gene expression assay method. The primers and probes for total human tissue factor (Hs00175225_m1) and for the control TATA box binding protein (TBP, Hs99999910_m1) were purchased from Applied biosystem. The primers and the probes for flTF (forward primer: TGATGTGGATAAAGGAGAAAACTACTGT, reverse primer: CTACCGGGCTGTCTGTACTCTTC, probe: FAM- TTCAAGCAGTGATTCCCTCCCGAACA–TAMRA); and asTF (forward primer: GGGATGTTTTTGGCAAGGACTTA, reverse primer: CCAGGATGATGACAAGGATGATG, probe: FAM-AATCTTCAAGTTCAGGAA AGAAATATTCTACATCATTGGA-TAMRA) were purchased from Eurogentec, Belgium [21]. The qPCR was performed by 10 min of initial denaturation and 44 cycles of 15 s at 95°C, 60s at 60°C in a CFX96® Real-time System (BioRad). Delta delta Ct method was used for analyzing qPCR results.

### Western blot

The cells were treated with 5–50 μM PD98059, 10 μM LY294002, 0.1 μM wortmannin, 10 μM A6730, 0.1 μM erlotinib, and 50 nM cetuximab or 30 nM of the mixture of siRNA for indicated time periods and washed 3 times. Cell lysates was obtained by incubating the cells in a lysis buffer [10 mM Tris pH 6.8, 1 mM EDTA, 10% NP40, 1 mM PMSF, 1% SDS plus protease inhibitor cocktail and phosphotase inhibitor cocktail 2 (Sigma) on ice for 30 min. Cell debris was removed by centrifugation at 16000 g for 10 min. Protein concentration was determined by a BCA^TM^ protein assay kit (Thermo Scientific, USA). Fifty microgram protein of each sample was loaded on 8% of SDS-PAGE gel for electrophoresis, and the protein was transferred onto polyvinylidene flroride (PVDF) membrane by the iBlot^TM^ dry blotting system (Invitrogen, USA). The membranes were blocked by 5% nonfat dry milk for 1 h and incubated with either monoclonal anti-TF antibody (# 4509, American Diagnostica, USA), or anti-phospho-Akt (Ser473) antibody or anti-Akt polyclonal antibody (# 9271 and 9272, cell signaling, USA) at 4°C for overnight, then the membranes were washed with Tris Buffered Saline-tween 20 (TBS-tween) buffer for 1 h and incubated with appropriate horseradish peroxidase (HRP)-conjugated secondary antibodies (Invitrogen, USA) diluted in blocking buffer for one hour at room temperature. After washing, western blotting luminol reagent (Santa Cruz Biotechnology, USA) was added onto the membranes and the chemiluminescence was recorded in a Fuji LAS-3000 system. Then the membranes were treated with antibody stripping buffer (Gene Bio-application Ltd. Israel), and incubated with anti-actin antibody (1:4000 dilution, Sigma, USA) and secondary antibodies for control. The results of western blot were quantified with Gel-Pro Analyzer software version 4.0.00.001 (Media Cybernetics, Bethesda, MD, USA).

### One stage clotting assay

MDA-MB-231 cells were treated for 24 h with 10 μM of PD98059, 10 μM of LY294002, 0.1 μM of wortmannin, or DMSO as control. Then the cells harvested with non-enzymatic detachment method were washed 3 times and suspended in barbital buffer at 10^5^/ml. Coagulant activity was tested at 37°C by adding 50 μl of cells to 100 μl of citrated normal plasma and 100 μl of 0.25 M CaCl_2_. The clotting time for each sample was determined from the addition of CaCl_2_ to the formation of first fibrin strand. The assay included a standard curve made with thromboplastin at the dilutions of 1:25 (100% activity), 1:50, 1:100, 1:200, 1:400, and 1:800. The cell membrane-associated TF activity determined with the clotting time was converted to thromboplastin procoagulant activity units (neoplastin, Stago, Asnières, France). The result is presented as percentage of the change in comparison with control cells. All the experiments were performed at least 3 times and each experiment was in triplicates.

### Matrigel invasion assay

MDA-MB-231 cells were treated with 10 μM of PD98059, 10 μM of LY294002, 0.1 μM of wortmannin or control DMSO for 24 h. The cells were washed 3 times and 0.5 ml of the cells (5 × 10^4^/ml) were seeded into each upper chamber of 24-well matrigel-coated invasion chamber and 0.75 ml DMEM supplemented with 5% fetal bovine serum was added into the lower chamber (BD Biosciences, USA). The invasion chambers were incubated for 22 h according to the manufacture’s protocol. Then the non-invading cells were removed from the upper surfaces of membranes by scrubbing with cotton tipped swabs and the cells on the lower surfaces of the membrane were fixed with 100% methanol and stained with 1% toluidine blue, the invading cells numbers were counted under an inverted microscope, and the invasion index were obtained by calculating the averages of the number of the cells per observation field of each sample in comparison with the control groups. The experiments was performed with 6 samples for one condition and repeated 3 times.

### Statistical analyses

Data were analyzed by Student’s *t*-test, one-way ANOVA and Mann–Whitney *U* test as appropriate. The data of qPCR and invasion assay are presented as mean ± SEM. The rest of data is presented as mean ± SD. A probability value ≤0.05 was regarded as significant.

## Results

### TF promoter activity down-regulated by PI3K pathway inhibitors and up-regulated by ERK inhibitor

To facilitate evaluating TF gene expression, we constructed a sub-cell line MDA-MB-231-TFluc, selected by antibiotic hygromycin resistance, which carries TF promoter that drives luciferase gene. The sub-cell lines showed a constitutive luminescence around 5×10^4^ channel numbers compared to the background levels of 30–50 channel numbers of the negative control parental cells.

PI3K inhibitors LY294002 and wortmannin, showed significant inhibitory effect on the TF promoter activity in MDA-MB-231-TFluc cells. As demonstrated in the decreased bioluminescent levels, TF promoter-driven luciferase activity was inhibited by both inhibitors (IC_50_ = 8.8 μM for LY294002 and IC_50_ = 0.12 μM for wortmannin) (Figure 1b, 1c). The inhibition of TF promoter activity was statistically significant and in a dose dependent manner for these two agents. Furthermore, the inhibitory effect of both agents was observed within the dose ranges of inhibitory activity as reported in the literature, showing that the effects were specific.

In contrast, ERK inhibitor PD98059 dramatically enhanced TF promoter-driving luciferase activity in the cells. A peak of activity was observed after 24 h treatment (Figure 1a). This enhancement was statistically significant, dose dependent and observed within the published dose range of its inhibitory effect on ERK.

### **TF mRNA and TF protein down-regulated by PI3K pathway inhibitors and up-regulated by ERK inhibitor**

**Figure 2:**
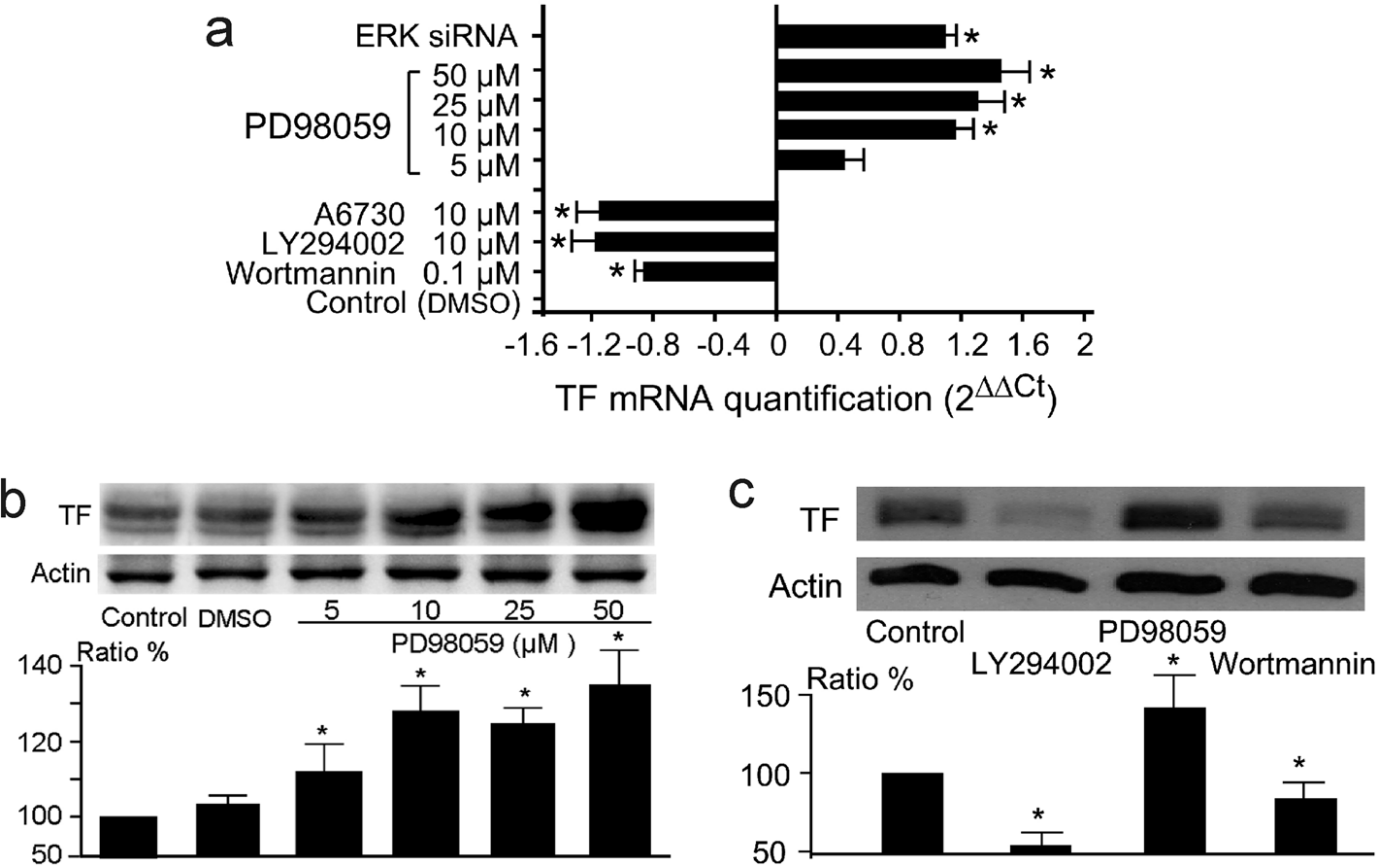
**Expression levels of TF mRNA and TF protein in treated MDA-MB-231 cells. Panel a:** The qPCR results of total TF mRNA levels in treated MDA-MB-231 cells. The cells were treated for 24 hr by the indicated agents at the indicated concentrations. qPCR was performed with primers Hs00175225_m1. The results were obtained from three independent experiments. Statistical significance (p<0.05) was found for all of the groups in comparison with the control group, except for the group of 5 μM PD98058. **Panel b:** The western blot of TF protein levels in PD98059-treated cells, showing a dose dependent increase in TF levels at 24 hrs. **Panel c:** The western blot of TF protein levels in the cells treated by LY294002 (10 μM) and wortmannin (0.1 μM) at 24 hrs. The data of the ratio were obtained with 3 repeated blots. * : p<0.05 in comparison with the controls.

According to the obtained results, MDA-MB-231 cells were treated with 10 μM LY294002 and 0.1 μM wortmannin. The qPCR and western blotting analysis showed that both LY294002 and wortmannin induced a remarkable decrease in TF mRNA and protein levels (Figure 2a,c). In contrast, PD98059 treatment enhanced dose-dependently tissue factor mRNA and protein levels in the cells (Figure 2a,b,c). qPCR assay with ERK siRNA confirmed the effect of PD98059 (Figure 2a). These results were well correlated with the data of luminescence assay.

### Blockage of PI3K/Akt pathway suppressed PD98059-induced high level of TF transcription

**Figure 3:**
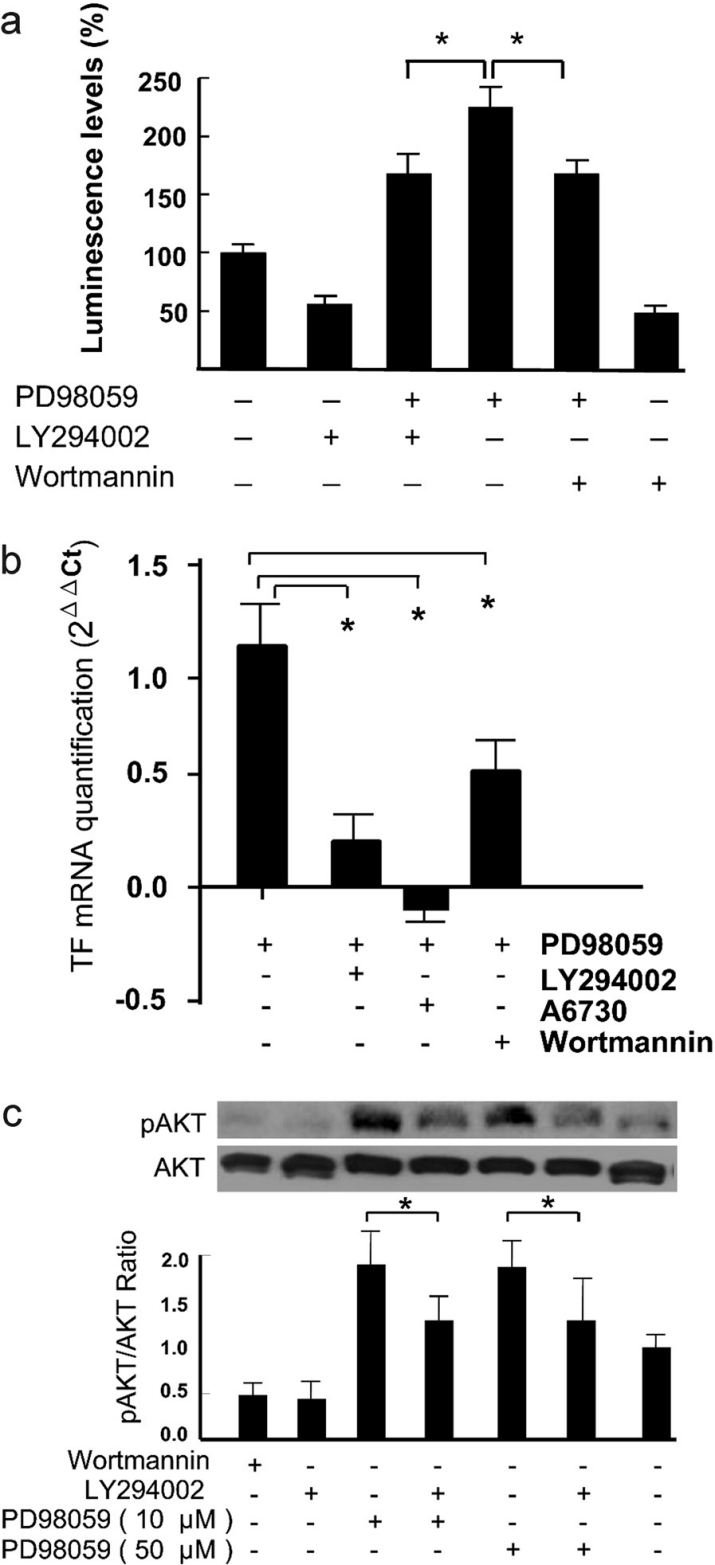
**TF promoter activity, TF mRNA and Akt phosphorilation after combined treatments. Panel a:** MDA-MB-231-TFluc cells were treated by 10 μM of LY294002 and 0.1 μM of wortmannin, in the absence or the presence of 10 μM PD98059 for 24 hrs. The activity of luciferase was measured. The data was represented as means±SEM of three independent experiments. * : p<0.05. **Panel b:** qPCR results of TF mRNA (Hs00175225_m1) in the cells after the treatment for 24 hrs by 10 μM of LY294002, 10 μM of A6730, 0.1 μM of wortmannin, in the absence or the presence of 10 μM PD98059. The experiments were repeated for 3 times. ** : p<0.05. **Panel c:** MDA-MB-231 cells were treated with 0.1 μM of wortmannin, 10 μM of LY294002 in the presence or the absence of PD98059 at 10 μM or 50 μM for 24 hrs. Total protein was extracted for the determination of pAkt level by western blot using anti-Akt or anti-pAkt antibodies. The data were from three blots. * : p<0.05.

We examined the relationship between PI3K and ERK pathways in the regulation of TF promoter in MDA-MB-231-TFluc cells. The MDA-MB-231-TFluc cells were treated by PD98059 in the presence of LY294002 or of wortmannin and the luminescence levels were determined. The results showed that both PI3K inhibitors could significantly suppress the PD98059-induced reporter gene expression (Figure 3a). Then MDA-MB-231 cells were treated in a similar way and their TF mRNA levels were quantified by qPCR method. The results showed that LY294002 and wortmannin as well as Akt inhibitor A6730 significantly suppressed PD98059-enhanced TF mRNA trnascription (Figure 3b). As Akt phosphorylation is a down-stream event of the activated PI3K, pAkt was checked in MDA-MB-231 cells. We found that pAkt level was enhanced by PD98059 treatment and this enhanced pAkt level was significantly inhibited by LY294002, showing a close relationship between ERK inhibition and Akt phosphorylation (Figure 3c).

### Involvement of EGFR in PD98059-enhanced high level of TF transcription

**Figure 4:**
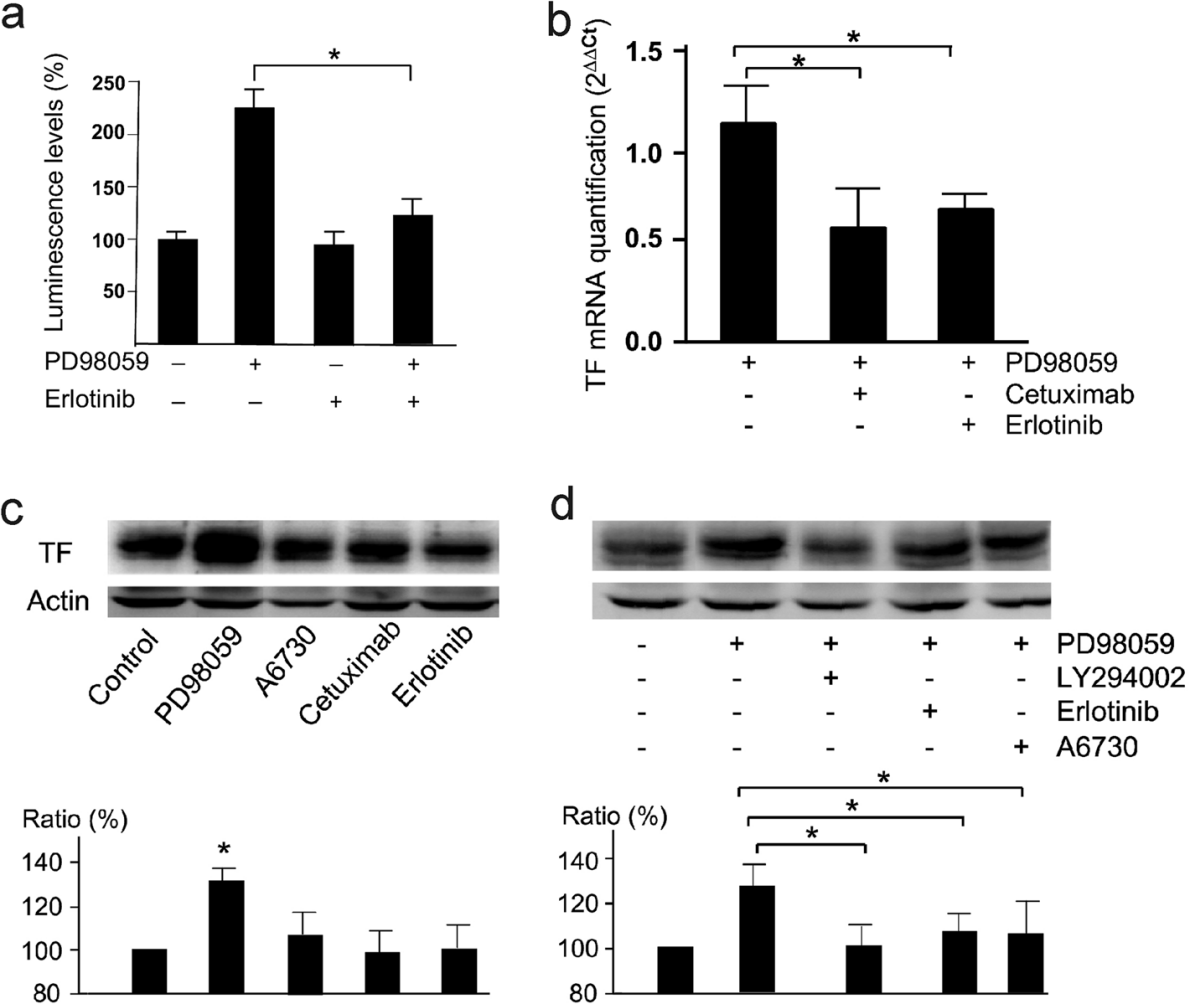
**TF promoter activity, TF mRNA and Akt phosphorilation after combined treatments. Panela:** MDA-MB-231-TFluc cells were treated by 0.1 μM of erlotinib in the absence or the presence of 10 μM PD98059 for 24 hrs. The cell activity of luciferase was measured. The data was from three independent experiments. **Panel b:** qPCR results of TF mRNA in the cells after the treatment for 24 hrs by 50 nM of cetuximab or 0.1 μM of erlotinib in the absence or the presence of 10 μM PD98059. The data were from 3 independent experiments. * : p<0.05. **Panel c:** The western blots of TF protein in the cells treated for 24 hrs by 10 μM PD98059, 10 μM of A6730, 50 nM of cetuximab and 0.1 μM of erlotinib. The data were from 3 blots. * : p<0.05 in comparison with control. **Panel d:** The western blots of TF protein in the cells treated for 24 hrs by 10 μM LY294002, 0.1 μM erlotinib and 10 μM A6730 in the presence or the absence of 10 μM PD98059. The data were from 3 blots. * : p<0.05.

Gan Y et al. have suggested that EGFR activity was involved in ERK inhibition-induced activation of PI3K/Akt pathway [22]. We used EGFR inhibitor erlotinib together with PD98059 to the culture of the MDA-MB-231-TFluc cells. The results confirmed the inhibition of PD98059-enhanced cell luminescence by erlotinib (Figure 4a). The results of qPCR also showed a significant inhibition of TF transcription by both erlotinib and anti-EGFR antibody cetuximab (Figure 4b). Western blot further confirmed the results of luminescence and qPCR by showing that erlotinib, like the inhibitors for PI3K/Akt suppressed significantly the PD98059-induced high level of TF protein synthesis (Figure 4c, d). We also noticed that erlotinib did not significantly affect TF protein level of the cells in culture without PD98059 induction. These data strongly indicated the involvement of EGFR was involved in PD98059-induced TF expression.

### Cell procoagulant and invading capacities correlated with TF expression

**Figure 5:**
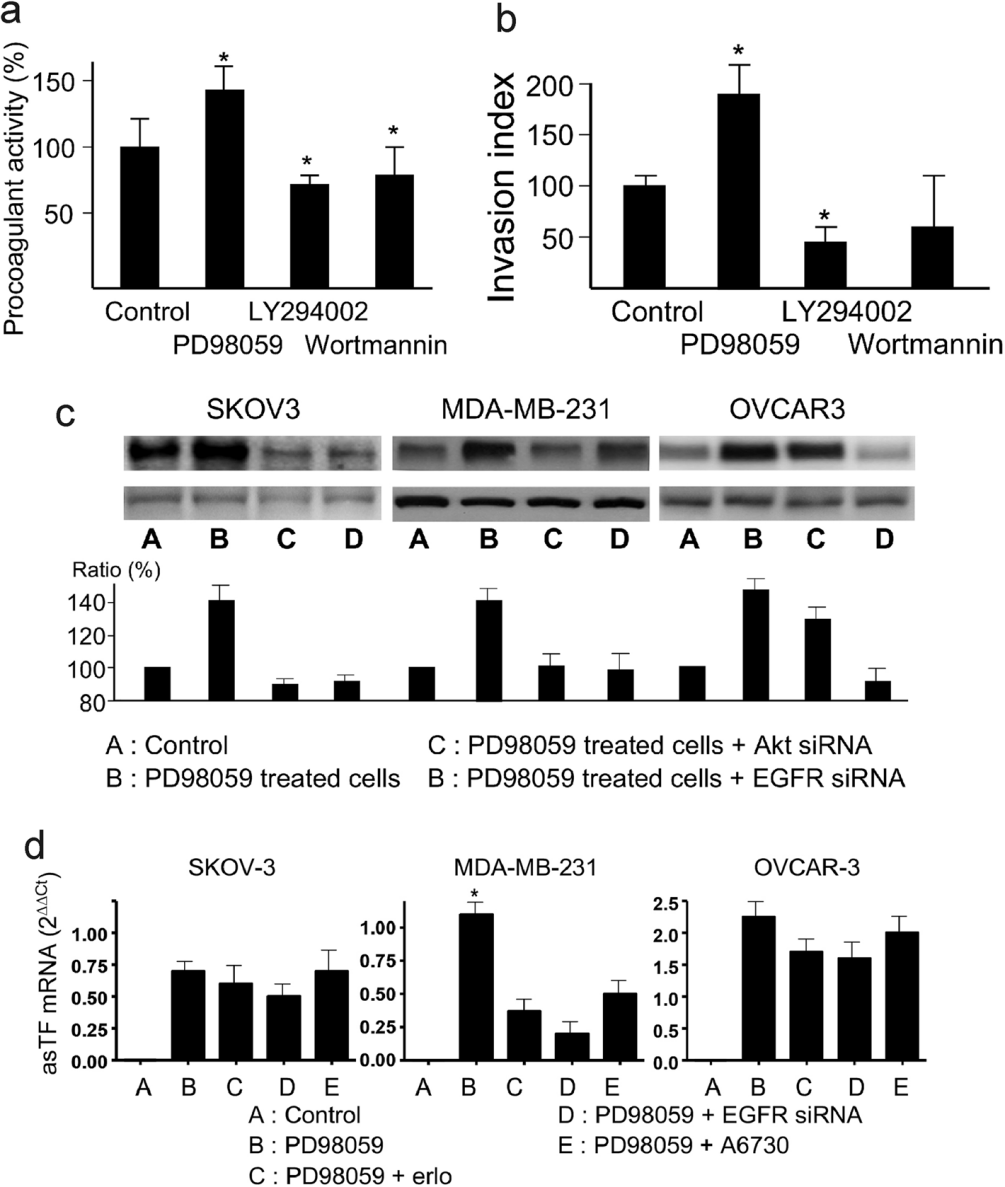
**Cell-associated procoagulant activity and invasion in MDA-MB-231 cells, flTF expression and asTF mRNA transcription in SKOV-3 and OVCAR-3 cells. Panel a: **Cell procoagulant activity. MDA-MB-231 cells were treated with 10 μM of PD98059, 10 μM of LY294002, 0.1 μM of wortmannin for 24 hrs and washed. Then clotting time was measured with these cells after the addition of human blood plasma and calcium. The results represent 3 different experiments. * : p<0.05 between PD98059 group versus LY294002 and wortmannin groups. **Panel b:** Cell invassive capacity : the same cells as in the panel a were cultured in the invasion chambers for 22 hrs. The cells penetrated through the matrigel were counted in microscopy. The data were from 3 independent experiments. * : P<0.05 in comparison to the control cells. **Panel c:** Regulation of TF protein in SKOV-3 and OVCAR-3 cells. SKOV-3 and OVCAR-3 cells were treated 10 μM of PD98059 with or without A6730 and EGFR siRNA for 24 hrs. The protein was extracted from the washed cells and examined for TF expression by western blot. The data were from 3 repeated blot experiments. Significant difference was found between the PD98059 group versus all of the other groups within the same cells (p<0.05). **Panel d:** asTF levels in PD98059 treated cells. The cells were treated by 10 μM of PD98059 with or without 0.1 μM erlotinib 10 μM A6730 and EGFR siRNA for 24 hrs. qPCR data were from 3 dependent experiments. * : p<0.05 for PD98059 groups in comparison with all of the other groups in the same cell lines.

To assess the relationship between the modulation of TF expression and cell-associated procoagulant activity, we performed one stage clotting assay with the microparticle-free MDA-MB-231 cells. We found that LY294002 and wortmannin inhibited the cell procoagulant activity, and that PD98059 induced an augmentation of the cell procoagulant activity (Figure 5a). These results indicated that the effect of LY294002, wortmannin and PD98059 on TF activity could be functionally relevant to cell’s pro-coagulant activity. They further suggested that the changes in TF expression on MDA-MB-231 might be related to the changes in cell invasion capacity through Matrigel matrix (Figure 5b).

### **TF regulation in SKOV-3 and OVCAR-3**

As EGFR was shown to be involved in the regulation of cell-associated TF expression, we performed the experiments of western blot with two EGFR positive cell lines SKOV-3 and OVCAR-3. The results showed that PD98059 upregulated TF synthesis in these two cell lines. Furthermore, Akt siRNA and EGFR siRNA suppressed PD98059-enhanced TF expression in these two cell lines in a similar manner to that in MDA-MB-231 (Figure 5c). We also performed qPCR to evaluate mRNA levels of asTF in these cells. asTF mRNA was found to represent about 2–5% of total TF mRNA. The results showed that PD98059 treatment stimulated asTF mRNA levels in all of the three cell lines, however, the blockage of Akt by A6730 and the blockage of EGFR by erlotinib and siRNA affected PD98059-enhanced asTF mRNA levels only in MDA-MB-231, but not in SKOV-3 and OVCAR-3 cells.

## Discussion

In this study, TF expression was studied with pharmacological inhibitors and siRNA that suppress PI3K/Akt and MAPK/ERK pathways [23]. Previous reports showed that these two pathways regulate both flTF and asTF transcription [15,24,25].

In agreement with other reports [7,8], an essential role of PI3K/Akt in TF expression in MDA-MB-231 cells was found because treatment by either LY294002 or wortmannin decreased TF expression in a dose-dependent manner. Experiments using Akt siRNA gave the same results. This was demonstrated by a decrease in the reporter gene expression using MDA-MB-231 cells transfected with the plasmid PGL4-TFluc as well as by qPCR using the parental cells. The decrease in TF gene expression was well correlated with the decrease in flTF protein and with the decrease in the cell surface-associated TF activity as shown by plasma clotting assays. We further showed that treatment with LY294002 and wortmannin resulted in inhibition of the catalytic activity of PI3K and Akt phosphorylation by western blot. All these findings confirmed that PI3K/Akt phosphorylation plays a critical role in TF gene expression.

In contrast to Akt inhibitors, we found that treatment with the ERK inhibitor PD98059 surprisingly resulted in a remarkable increase in TF gene expression in a dose-and time-dependent manner. This finding was initially observed in MDA-MB-231-TFluc cells, and then confirmed by qPCR and western blot with their parental cells. The use of ERK siRNA further confirmed this observation. Therefore, Akt and ERK modulated TF expression in opposite ways.

To study the mechanisms involved, we blocked PI3K/Akt activation by LY294002, wortmannin, A6730 or Akt siRNA in PD98059-treated MDA-MB-231 cells. These experiments gave concomitant results showing that PD98059-induced TF expression was indeed inhibited at mRNA and protein levels by blocking the PI3K/Akt pathway, and in particular, the blockage was complete using Akt inhibitor A6730. These results emphasized the importance of the PI3K/Akt pathway in the control of TF expression.

In the literature, many studies reported the interaction of growth factor receptors with ERK and PI3K/Akt pathways and the crosstalk between ERK and PI3K/Akt pathways [26-31]. Gan et al. demonstrated that blockage of ERK activity enhanced EGF receptor activation and turnover, which in turn enhanced PI3K activation and Akt phosphorylation [22]. For this reason, we explored the role of EGFR in the PD98059-induced TF up-regulation. Our results from qPCR and western blot experiments showed that the EGFR inhibitor erlotinib indeed suppressed PD98059-induced TF expression. We also observed that the inhibitory effect of erlotinib was much more noteworthy in PD98059-treated cells than in non-treated cells. The experiments using EGFR siRNA gave similar results. These results strongly suggest that the similar regulation described by Gan et al. occurred in MDA-MB-231 cells [22]. In brief, the inhibition of ERK activity by PD98059 enhanced EGFR activity, which in turn up-regulated Akt activity, resulting in high levels of TF expression. Such a mechanism can explain how the blockage of ERK induced a high level of TF expression, and why blockage of the Akt pathway suppressed such an induction. The same profile of TF regulation was again observed in OVCAR-3 and SKOV-3 cells, suggesting a widespread mechanism. Our results do not exclude other signal interconnections and we believe that the full mechanism of TF regulation is likely more complicated and further study is needed. Our results contradict a previous report showing inhibition of TF expression by ERK inhibitor [9], however, the reason for this discrepancy is unclear.

As the inhibition of PI3K/Akt may reduce asTF mRNA in endothelial cells [15], we evaluated the asTF isoform in response to the addition of inhibitors of PI3K/Akt and MAPK/ERK. We observed in MDA-MB-231, SKOV-3 and OVCAR-3 cells that PD98059 up-regulated asTF. However, the inhibition of PD98059-enhanced asTF mRNA transcription by Akt inhibitors was observed only in MDA-MB-231. The results of the asTF mRNA levels in SKOV-3 and OVCAR-3 cells seem to suggest that asTF level could also be regulated independently from flTF expression [15]. They indicate the complexity of the regulation of TF isoform transcription. Further investigation is needed to clarify these. Our observation in MDA-MB-231 also suggests that the increase in the membrane-associated flTF and in the secretion of asTF can occur concomitantly during malignant transformation. flTF is known to stimulate tumor progression via FVIIa and PAR2 and asTF has been shown to induce tumor angiogenesis by its binding to integrins [16]. The level of asTF was found to be related to poor clinical prognostics [17]. The secretion of asTF by cancer cells has been shown to be a complex process which is under the control of SR proteins in addition to TF promoter and miRNA regulation [15,31], Further investigation can be expected to better understand the regulation of TF including its isoforms in detail. Our results do not exclude a distinct SR protein-mediated regulatory mechanism for asTF production which has been reported to be independent from transcriptional regulation for TF [15].

Our results support and underline the roles of Akt and EGFR in TF-related tumor growth and metastasis. We believe that targeting TF expression could potentially improve clinical cancer therapy by inhibiting tumor angiogenesis and metastasis as well as by controlling thrombotic complications [32,33].

## Conclusions

This study showed a regulatory mechanism in which MAPK/ERK signals inhibit EGFR/PI3K/Akt-mediated TF expression in breast cancer MDA-MB-231 cells. The same regulation was observed in ovarian cancer OVCAR-3 and SKOV-3 cells. We also showed that both flTF and asTF could be regulated in a parallel manner. As the PI3K/Akt pathway and EGFR regulate TF expression in cancer cells, targeting these signaling components is expected to potentially inhibit TF expression-associated tumor progression.

## Abbreviation

MAPK, Mitogen-activated protein kinase/Extracellular signal-regulated kinase; ERK, Extracellular signal-regulated kinases; PI3K, Phosphoinositide 3-kinase; AKT, Akt1 or protein kinase B (PKB); siRNA, Small interfering RNA; EGFR, Epidermal growth factor receptor; qPCR, Quantitative polymerase chain reaction.

## Competing interests

The authors declare that they have no competing interests.

## Authors’ contributions

HL, HL, CS,AJ and XX conceived of the study, participated in the design of the studyand HL, HL, CS, CH and LH drafted the manuscript. CH, LH, CG performed the experimental studies. All authors read and approved of the final manuscript.
